# Draft Genome Sequence of a Papaverine-Degrading, Gram-positive *Arthrobacter* sp., Isolated from Soil Near Hohenheim, Germany

**DOI:** 10.1128/genomeA.00422-15

**Published:** 2015-05-21

**Authors:** Ondrej Reznicek, Sandra J. Facey, Bernhard Hauer

**Affiliations:** Institute of Technical Biochemistry, University of Stuttgart, Stuttgart, Germany

## Abstract

We present the 4.8-Mb draft genome of a soil bacterium identified as *Arthrobacter* sp. This Gram-positive soil bacterium is able to use the aromatic compound papaverine as sole carbon source and will be examined for novel oxygenases.

## GENOME ANNOUNCEMENT

Using papaverine as a substrate, a soil bacterium was isolated in Hohenheim, Germany, in the late 1970s (1). Morphological and physiological tests identified this microorganism as *Nocardia* sp. During cultivation on papaverine as the sole source of carbon and nitrogen, several substrate intermediates were found to be excreted into the medium. A partial degradation pathway for papaverine involving initial ring hydroxylation was proposed (1, 2). Here, we present a draft genome sequence by shotgun sequencing to explore its enzymatic content for novel oxygenases capable of converting bulky aromatic substrates such as papaverine.

The draft genome of this microorganism was obtained using Illumina shotgun and mate-pair sequencing, which resulted in paired-end reads of 250 bp. Raw data, consisting of 9,583,484 reads, were clipped and trimmed with CASAVA Illumina software. Reads with more than one *N* or a final length of <20 bases were removed. Quality trimmed reads were error-corrected using Musket version 1.0.6, with a 21-bp *k*-mer size for correction. The error-corrected reads were digitally normalized by normalize_by_median.py from the “khmer” package version 0.3, with a coverage cutoff from 80, and reads with <21 bases were discarded, yielding a data set of 2,365,326 reads. Allpaths LG release 47547 software was used to assemble the digitally normalized reads into scaffolds. Gap closure and refinement of the scaffolds was done with SOAP GapClosure version 1.12 and SEQuel version 1.0.2, respectively. All reads were aligned against the assembled scaffolds with Bowtie2 version 2.1.0. Assembly of the digitally normalized reads resulted in three genomic scaffolds in total, with a GC content of 62%. Among the three, scaffold_1 was the largest (2.83 Mb), followed by scaffold_2 (1.1 Mb) and scaffold_3 (917 kb).

For taxonomic classification, phylogenetic analysis based on the 16S rRNA sequence within scaffold_1 was performed using the eubacteria primers 8F (5′ AGAGTTTGATCCTGGCTCAG 3′) and 1492R (5′ CGGTTACCTTGTTACGACTT 3′). An NCBI BLAST search showed an absolute sequence identity of the formerly identified *Nocardia* sp. as now related to the genus *Arthrobacter*. This new classification was additionally confirmed with a BLAST search within the EzTaxon-e databank (3).

The Glimmer program (4) was used to search for open reading frames (ORFs) coding for oxygenases within the scaffold sequences. The ORFs were blasted against the NCBI nonredundant, Gen3D, SMART, Pfam, TIGRFam, SUPERFAMILY, HAMP, PIRSF, COILS, and InterProScan databases. Out of a total of 4,599 proteins, the global annotation revealed 11 monooxygenases and 4 α-subunits of Rieske nonheme iron aromatic ring-hydroxylating oxygenases (RHOs). Such RHOs are prevalently responsible for microbial degradation of aromatic compounds (5, 6) and have been proposed to be involved in this bacterium’s initial conversion of papaverine. Being one of the first isolated soil bacteria capable to degrade papaverine as a complex aromatic substrate for energy supply, further investigation will be done to characterize these oxygenases in more detail for application in biotechnology (7) and organic synthesis.

### Nucleotide sequence accession numbers. 

The scaffolds of this whole-genome shotgun project has been deposited in the European Nucleotide Archive (ENA) under the accession numbers CVLG01000001 to CVLG01000003.
